# Antioxidative, Anti-Inflammatory, Anti-Obesogenic, and Antidiabetic Properties of Tea Polyphenols—The Positive Impact of Regular Tea Consumption as an Element of Prophylaxis and Pharmacotherapy Support in Endometrial Cancer

**DOI:** 10.3390/ijms23126703

**Published:** 2022-06-16

**Authors:** Piotr Olcha, Anna Winiarska-Mieczan, Małgorzata Kwiecień, Łukasz Nowakowski, Andrzej Miturski, Andrzej Semczuk, Bożena Kiczorowska, Krzysztof Gałczyński

**Affiliations:** 1 Department of Gynecology and Gynecological Endocrinology, Medical University of Lublin, Aleje Racławickie 23, 20-049 Lublin, Poland; piotrolcha@op.pl; 2 Department of Bromatology and Nutrition Physiology, Institute of Animal Nutrition and Bromatology, University of Life Sciences in Lublin, Akademicka 13, 20-950 Lublin, Poland; malgorzata.kwiecien@up.lublin.pl (M.K.); bozena.kiczorowska@up.lublin.pl (B.K.); 3 Department of Gynecology, 1st Clinical Military Hospital in Lublin, Al. Raclawickie 23, 20-049 Lublin, Poland; luknowakow@gmail.com (Ł.N.); a.miturski@gmail.com (A.M.); 4 Second Department of Gynecology, Medical University of Lublin, Jaczewskiego 8, 20-954 Lublin, Poland; andrzejsemczuk@umlub.pl; 5 Faculty of Medical Sciences and Health Sciences, Siedlce University of Natural Sciences and Humanities, Konarskiego 2, 08-110 Siedlce, Poland; krzysztof.galczynski@gmail.com

**Keywords:** endometrial cancer, tea consumption, tea polyphenols, prophylaxis and pharmacotherapy support, anti-obesogenic, antidiabetic, antioxidative effect

## Abstract

Endometrial cancer (EC) is second only to cervical carcinoma among the most commonly diagnosed malignant tumours of the female reproductive system. The available literature provides evidence for the involvement of 32 genes in the hereditary incidence of EC. The physiological markers of EC and coexisting diet-dependent maladies include antioxidative system disorders but also progressing inflammation; hence, the main forms of prophylaxis and pharmacotherapy ought to include a diet rich in substances aiding the organism’s response to this type of disorder, with a particular focus on ones suitable for lifelong consumption. Tea polyphenols satisfy those requirements due to their proven antioxidative, anti-inflammatory, anti-obesogenic, and antidiabetic properties. Practitioners ought to consider promoting tea consumption among individuals genetically predisposed for EC, particularly given its low cost, accessibility, confirmed health benefits, and above all, suitability for long-term consumption regardless of the patient’s age. The aim of this paper is to analyse the potential usability of tea as an element of prophylaxis and pharmacotherapy support in EC patients. The analysis is based on information available from worldwide literature published in the last 15 years.

## 1. Introduction

Globally, endometrial cancer (EC) ranks as the second, after cervical cancer, most commonly diagnosed malignant tumours affecting female reproductive organs, and the sixth most common cancer diagnosed in the female population overall. In 2020 alone, 9,227,484 new malignant tumour cases were diagnosed in women worldwide, of which nearly 420,000 or 4.5% were cases of EC. The corresponding number of recorded deaths attributed to cancer in this period was 4,429,323, with 97,370 associated with EC. It is estimated that 8.7/100,000 women will suffer from EC [1]. In highly developed countries, EC incidence ranks the fourth highest after breast, lung, and colorectal cancer. The highest EC incidence and mortality rates are observed in North America and central and eastern Europe, where the ASR (age-standardised rate) is, respectively, 21.1/100,000 and 20.2/100,000 women; the lowest rates are reported in central Africa and southern and central Asia, respectively, 2.3 and 2.7/100,000 women [1]. Among women living in the US, malignant endometrial cancer is responsible for an average of 7%, and in Europe, for 6.2%, of all malignant tumours diagnosed [2]. The highest incidence of EC in European countries has been reported in Belarus, Slovakia, Latvia, and the Czech Republic (respectively, 19.8, 19.4, 19.1, and 18.1/100,000 women) [3]. A vast majority of EC cases are diagnosed in women over 50, and the average age of diagnosis is 63 [1]. The average likelihood of contracting EC over the course of one’s entire life is approximately 2% [4,5]. The rate of EC-related deaths also increases with age. Endometrial cancer mortality in patients aged between 19 and 39 years is 0.1 in 100,000, whereas in patients aged 55–59 years, it increases to 5.2/100,00, and in patients over 75 years old, it is already 18.5/100,000 [1].

**Figure 1:**
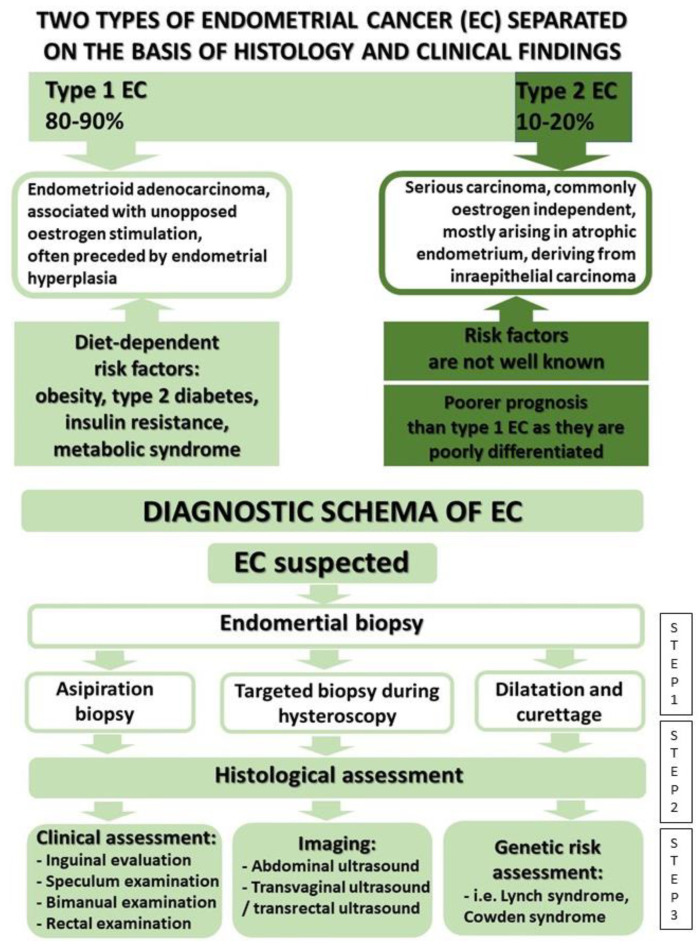
Types of EC and diagnosis schema.

Two types of endometrial cancer can be distinguished based on significant differences in terms of treatment success and patient survival (Figure 1).

Type 1 cancer, diagnosed in 80% of the cases, is described as endometroid and includes cases of highly and intermediately differentiated cancer (G1, G2) [6]. It develops from proliferation caused by the stimulation, not balanced by progesterone or other progestogens, of the endometrium by endogenic (similarly to obesity) or exogenic oestrogens (during systemic monotherapy in women with a preserved uterus). Other risk factors relevant to this type of cancer include childlessness, early menarche and late menopause, long-term therapy with drugs from the group of selective oestrogen receptor modulators, diabetes, hypertension, and certain genetic syndromes [7,8,9]. Type 2 endometrial cancer is responsible for the remaining 20% of cases and includes poorly differentiated endometrioid cancers (G3) as well as serous, clear-cell, mucinous, squamous, and other tumours. It is characterised by an aggressive clinical course and an unfavourable prognosis [6].

**Figure 2:**
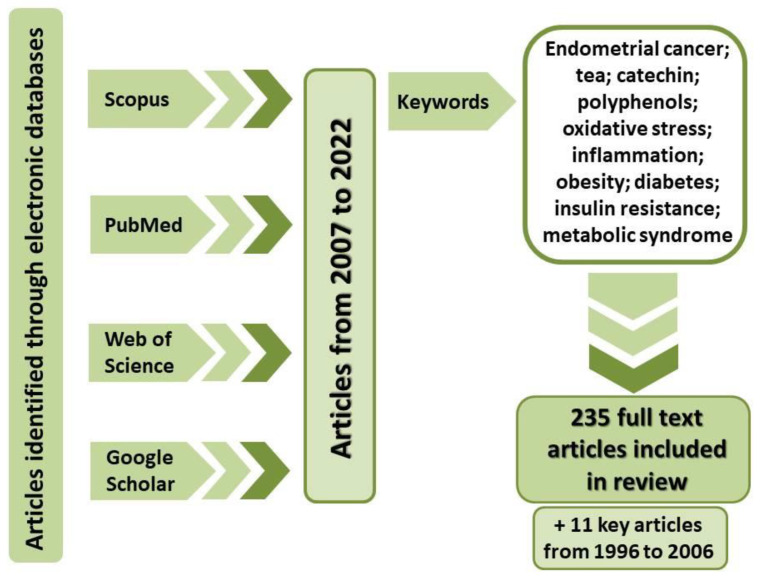
Literature search strategy.

Diet-dependent factors influencing the development of EC include obesity, type 2 diabetes and/or insulin resistance, as well as metabolic syndrome [7]. They are all diseases whose physiological markers entail functional disorders of the antioxidant system and progressing inflammation [10,11,12]. Moreover, given the fact that the physiological markers of EC also include antioxidative system disorders and inflammation [13,14,15], the primary form of dietary pharmacotherapy supplementation should involve foods rich in substances aiding the organism’s response to this type of disorder, in particular ones that are suitable for lifelong consumption. Tea satisfies those requirements thanks to its proven antioxidative, anti-inflammatory, anti-obesogenic, and antidiabetic properties. The aim of this paper is to analyse the potential usability of tea as an element of prophylaxis and pharmacotherapy support in EC patients. The analysis is based on information available in worldwide literature published in the last 15 years, using the following databases: Web of Science, Scopus, PubMed, and Google Scholar (Figure 2). The information analysis was conducted in January and February 2022.

## 2. Genetic Factors

The available literature provides evidence for the involvement of 32 genes in the hereditary incidence of endometrial cancer, identified in the course of a quasi-populational analysis of patients suffering from EC [16]. In type I EC (oestrogen-dependent), the most common symptoms include disorders in the expression of progesterone and oestrogen receptors, mutations of the PTEN (10q23.3) and KRAS (12p12.1) genes, and microsatellite instability, whereas in type II EC (hormonally independent), the most common symptoms include mutations of HER2/neu (17q12–21.32) and TP53 (17p13.1) genes [17]. Saule et al. [18] observed a potential relationship between EC (serous) and the presence of mutated BRCA1 (17q21) genes. Reports also mention a germinal mutation of the POLD1 (19q13.33) gene responsible for encoding the polymerase catalytic subunit DNA δ constituting an EC-specific molecular subgroup [9]. Somatic mutations of the POLE (12q24.3) gene are reported in 6–10% of EC cases [19].

In a cohort study conducted in a group of 11,847 carriers of the BRCA1 variant, a two- or three-fold increase in the incidence of EC was reported, but it should be considered that BRCA1 variant carriers can receive tamoxifen which exacerbates the risk of EC [20]. The relationship between BRCA1 mutations, EC, and tamoxifen has also been reported in other studies [21,22].

With the risk factor of 1.5 to 2.1, a higher incidence of EC is observed in women with a history of the disease diagnosed in a first-degree relative, as evidenced in case-control studies [23,24]. Additionally, in a study by Win et al. [24], it was demonstrated that in women with a history of EC or colorectal cancer among first-degree relatives, the risk of EC is higher than in women with no such history.

Lynch syndrome is the most common genetic syndrome associated with EC, as well as a significantly higher risk of hereditary non-polyposis colorectal cancer unrelated, which is why some authors suggest the need to screen EC patients for Lynch syndrome [8]. In patients with Lynch syndrome coexisting with EC, mutations of DNA mismatch repair (MMR) genes, including MSH2 (2p21-p16.3), MLH1 (3p22.2), and MSH6 (2p16.3), have been observed [25,26]. In women with EC and Lynch syndrome, the incidence of mutations of the MSH2 gene is 50–66%, of MLH1 24–40%, and of MSH6 10–13% [27]. In such individuals, mutations also occur in the KRAS, PTEN, and PIK3CA (3q26.32) genes, as well as in β-catechin involved in cellular adhesion [28,29,30]. The likelihood of EC in women suffering from Lynch syndrome is between 40 and 60% [31]. In another study conducted in Canada, it was reported that Lynch syndrome was diagnosed in 6.4% of EC patients over the age of 70 [32].

Cowden syndrome is a rare condition typically caused by a mutation in the PTEN tumour suppressor gene (10q23.3) present in 85% of sufferers [33]. The gene encodes the photostase involved in cellular signalling pathways affecting cell proliferation and survival [9]. In approximately 10% of Cowden syndrome patients, the PTEN variant is absent; instead, the presence of germinal variants is detected in AKT1 (14q32.33) or PIK3CA, i.e., the genes actively involved in the same PI3K/AKT signalling pathway that is inhibited by PTEN [34]. The risk of contracting EC in Cowden syndrome patients is 20–28% [9,35].

## 3. Treatment

The treatment of EC involves surgery, radiotherapy, radio-chemotherapy, hormonal therapy, and chemotherapy. The recommended ratio of surgery, radiotherapy, chemotherapy, and hormonal therapy in the treatment of primary endometrial cancer is, respectively, 84%, 46%, 20.8%, and 0.2% [36]. Surgery undergone by EC patients entails hysterectomy and salpingo-oophorectomy and the removal of the iliac and paraaortic lymph nodes (lymphadenectomy). The procedure is performed by laparoscopy or laparotomy. Lymphadenectomy is key to the successful surgical treatment of EC. The lymph drained from the organs in the pelvis flows down to the iliac, obturator, presacral, and perianal lymph nodes. Histopathological analysis of the dissected nodes allows practitioners to assess the clinical stage of the cancer and plan further treatment. Additionally, if metastasised lymph nodes are removed, the surgery can serve an important therapeutic role. It is recommended that when determining the stage of endometrial cancer, the removed lymph nodes should include common, external, internal iliac, and obturator lymph nodes. Additionally, the lymphadenectomy ought to be complemented by the removal of paraaortic nodes up to the renal vein [37,38,39]. The presence or absence of extrauterine foci determines the specific treatment regimen and patient follow-up, as well as the prognosis. In cases of poorly differentiated EC or if infiltration of the myometrium is detected, lymphadenectomy is obligatory. In cases of highly differentiated cancer or if the disease is limited only to the endometrium, the need for systemic lymphadenectomy is debatable. There are several concepts of pelvic lymphadenectomy. Iliac lymph nodes can be removed completely (total lymphadenectomy of the pelvis), selectively, or only if a macroscopic and palpable examination reveals lesions. An increasingly common practice entails identifying the sentinel lymph node whose histopathological evaluation is key to determining the course of the subsequent treatment. The sentinel lymph node is the one directly receiving lymph via the lymphatic vessels originating from the tumour. In the case of metastasis, this will be the first involved lymph node. The identification of the sentinel lymph node in the staging of endometrial cancer is currently becoming an increasingly common strategy. The examination of the sentinel lymph node can be crucial to the decision regarding subsequent lymphadenectomy. If no metastasis is detected in the lymph node, systemic lymphadenectomy is not required, which reduces the risk of associated complications and significantly shortens the duration of the surgery. The detection of the sentinel lymph node also prevents issues related to situations where the node is located outside of the standard area of lymphadenectomy. The most popular methods of identifying the sentinel lymph node entail the use of indocyanine green, methylene blue, or radioisotopes. These are typically applied in the region of the uterine cervix. The method involving detection of the sentinel lymph node during a laparoscopic procedure is consistent with the standard of minimising treatment invasiveness. Nowadays, indocyanine green particularly tends to be the most popular choice in the context of mapping sentinel lymph nodes [40,41].

Although EC is the most common malignant tumour of the female reproductive organs, it remains exceedingly rare in young women and teenagers. Obesity, chronic anovulation caused by polycystic ovary syndrome, and hereditary neoplastic syndromes such as Lynch or Cowden syndrome are all confirmed risk factors observed in young patients affected by the cancer. The growing incidence of obesity in children and teenagers is likely responsible for the increasing number of EC cases and the related preneoplasia observed in younger individuals. The consequences of endometrial cancer can be catastrophic in terms of fertility as the standard treatment protocol entails excision of the uterus with uterine adnexa. Particular diagnostic care should be exercised in teenage patients affected by the aforementioned risk factors who experience abnormal vaginal bleeding. The early diagnosis of the cancer or its precursors is of crucial importance and may potentially facilitate a fertility-sparing approach. Such a therapy entails the oral administration of progestogens or the use of an intrauterine device releasing levonorgestrel. Gonadotropin-releasing hormone (GnRH) analogues or aromatase inhibitors have also been observed to be effective in the treatment of early-stage and highly differentiated tumours. The applicability of fertility-sparing treatment is limited only to patients with a low-risk disease [42,43].

## 4. Oxidative Stress in Endometrial Cancer

Reactive oxygen species (ROS) react with polyunsaturated fatty acids present in cellular membranes, which triggers the process of lipid peroxidation leading to protein modifications, changes to the membrane gradient and, consequently, a loss of membrane integrity and irreparable damage [44,45]. Elevated ROS content in cells may result from a weakening of the organism’s antioxidative mechanisms, primarily due to reduced concentrations of reduced glutathione (GSH), the total pool of –SH groups related to proteins, and changes in terms of antioxidative enzyme activity [45]. Oxidative stress leads not only to inflammatory response as such but also to the activation of an NF-κB protein-dependent (nuclear factor-κB) transcription of genes for various inflammatory factors [46]. Strong oxidative stress can play an important role in carcinogenesis as it causes damage to and mutation of tumour suppressor genes [47]. High concentrations of ROS in tumour cells may also induce cellular adaptation, increased rates of proliferation, DNA mutations and genome instability, as well as the development of resistance to certain groups of drugs used in cancer therapy, which further stimulates tumour growth [48]. Similarly to other cancers, EC entails chronic oxidative stress (Table 1), primarily due to the development of inflammation and the activity of cytokines, as well as oncogenic signalling, intensive metabolism due to continuous proliferation, mutations in the mitochondrial DNA, and dysfunctions of the respiratory chain [49].

The overproduction of cytokines resulting from chronic inflammation may induce neoplastic transformation and, subsequently, facilitate tumour development through the expression of cytokines by cells present in the tumour’s microenvironment [50]. ROS-induced chemical modification of genetic material is the first step in the process of mutagenesis and carcinogenesis [50]. Damaged DNA can cause changes to the process of transcription, replication errors, induction of signal transduction pathways, and genome instabilities, i.e., the basis of carcinogenesis. The formation of a tumour is a multi-stage process that includes the phases of initiation, promotion, and progression. As follows from numerous studies conducted in experimental in vivo and in vitro systems, ROS overproduced under the conditions of oxidative stress are involved not only in the stages of initiation and promotion but also in the progression of carcinogenesis [47]. ROS are among the factors responsible for inducing the neoplastic transformation of cells and play a two-fold role in neoplasia: on the one hand, they can promote pro-tumorigenic signalling, facilitating the proliferation of tumour cells, their survival and adaptation to hypoxia, and on the other, they can promote anti-tumorigenic signalling and cause the death of tumour cells due to oxidative stress [51].

It has been observed that antioxidative activity is reduced with increasing levels of oxidative stress markers in women with early-stage EC [13]. An analysis of the prognostic values of oxidative stress parameters (oxidative stress index OSI, total oxidant status TOS) and M30/65 apoptotic markers concentration in the blood serum of 52 women diagnosed with EC indicated that the markers were significantly higher in the sick patients, as compared to the control group, while the levels of antioxidative markers (total antioxidant status—TAS) in the serum were significantly lower [14]. Although impaired apoptotic activity plays a key role in EC aetiopathogenesis, oxidative stress may be a major factor in the malignant transformation. Oxidative stress exacerbates both the development and progression of the disease, while the imbalance of thiol disulphide homeostasis caused by oxidative stress may be a contributor to EC aetiopathogenesis, as demonstrated in a study conducted in a group of 57 EC patients [15]. In the cited study, the blood serum of the affected women was observed to contain statistically decreased concentrations of negative thiols and total thiols. The intra- and extracellular redox states of thiols are crucial to the structural and functional condition of proteins, the regulation of enzyme activity, management of transcription factors’ activity, and antioxidative defence [52]. The compounds constitute a component of the thiol-disulphide redox buffer; they act as sweepers of free radicals and chelators of prooxidative metals. In order to prevent cell damage caused by free radicals, the organism develops dedicated defence mechanisms; the endogenic antioxidative system utilising antioxidative enzymes: superoxide dismutase (SOD), catalase (CAT), glutathione peroxidase (GSHPx), and glutathione reductase (GR), as well as low-molecular antioxidants such as glutathione and plasma proteins, and antioxidative vitamins: E, C, and A [52,53].

**Table 1:** Antioxidant capacity in endometrial cancer.

Oxidative Stress Parameters	Target Sites	References
↑ TOS; ↓ TAC	Serum	[13]
↑ MDA, ↑ vit. E., ↑ TBARS	Serum	[54]
↑ LOOHs, ↓ PON-1, ↑ Vit. E	Serum	[55]
↑ Vit. E, ↓ SOD	Serum	[56]
Positive correlation between increasing BMI and markers of oxidative stress; positive correlation between cancer and the percent of GSH as GSSG in the samples run as cancer vs. non-cancer	Serum	[57]
No correlation between PON-1 and the stage of the disease	Serum	[58]
↑ CAT, ↑ Cu, ↑ ceruloplasmin	Serum	[59]
↓ TAS, ↓ native thiol	Serum	[60]
↑ glutathionylated protein, ↓ GSH	Serum	[61]
↑ TOS, ↑ OSI, ↑ TAS	Serum	[14]
↓ SOD, ↑ NO, ↓ CAT	Serum	[48]
↓ ORAC, ↓ Vit. C, ↓ Vit. E, ↓ Vit. A, ↑ GSH, ↑ SOD	Serum	[62]
↑ Vit. A; ↓ Vit. E; ↓ Vit. C	Serum	[63]
↓ GSH, ↑ MDA, ↑ SOD, ↑ 8-OHdG	Serum	[64]
↑ SOD, ↓ GSH, ↓ GSH-Px	Serum	[65]
↑ SOD	Serum	[66]
↑ MDA, ↓ TAC	Serum	[67]
↑ SOD, ↑ 8-OHdG, ↓ GSH-Px, ↑ 8-OHdG/GSH-Px	Serum	[68]
↑ GSH-Px; ↑ SOD	Serum	[69]
↑ AOPP, ↑ nitrates and nitrites; significant correlation between pelvic pain symptom scores and peritoneal protein oxidative stress markers	Peritoneal fluid	[70]
↑ ox-LDL, ↑ 8-OHdG, ↑ 8-isoprostane, ↑ 8-isoPGF2α, ↑ 25-hydroxycholesterol	Peritoneal fluid	[71]
↑ MDA, ↑ LOOHs	Peritoneal fluid	[72]
↑ 8-OHdG, ↑ 8-isoprostane, ↑ 8-isoPGF2α, ↑ 25-hydroxycholesterol	Peritoneal fluid	[73]
↑ 8-OHdG	Ovarian cortex tissue	[74]
↑ LPO, ↓ TAC	Follicular fluid	[54]
↑ ROS, ↑ MDA, ↑ NO, ↓ SOD, ↓ CAT, ↓ GSH-Px, ↓ Vit. A, ↓ Vit. C, ↓ vit. E	Follicular fluid	[75]
↓ SOD, ↓ CAT, ↓ GSH-Px, ↓ GR/GSH, ↓ Vit. A, ↓ Vit. C, ↓ Vit. E	Follicular fluid	[56]
↑ 8-isoPGF2α	Urine	[76]
↓ 8-OHdG	Urine	[77]
↓ GSH; ↓ SOD	Tissues gums	[78]

↑—increased concentration or activity compared to control group; ↓—decreased or inhibited concentration or activity compared to control group; 8-OHdG—8-hydroxy-2-deoxyguanosine; 8-isoPGF2α—8-izoprostoglandyna-F2α; AOPP—advanced oxidation protein products; BMI—body mass index; CAT—catalase; Cu—copper; GSH—glutathione; GSH-Px—glutathione peroxidase; GSSG—oxidized glutathione; GR—glutathione reductase; LOOHs—lipid hydroperoxides; LPO—lipid Peroxidation; MDA—malondialdehyde; NO—nitric oxide; NOS 2—nitric oxide synthase 2; OSI—oxidative stress index; ORAC—total antioxidant status; ox-LDL—oxidized LDL; PON-1-paraoxonase-1; ROS—reactive oxygen species; SOD—superoxide dismutase; TAC—total antioxidant capacity; TAS—total antioxidant system; TBARS—thiobarbituric acid reactive substances; TOS—total oxidant status; Vit. A—vitamin A; vit. C—vitamin C; vit. E—vitamin E.

An additional element of the oxidative stress defence system is constituted by paraoxonase-group enzymes (PON), including paraoxonase I, II, and III. In the event of deficiency in terms of any of the system’s elements, the cellular antioxidative potential is lowered, which impairs the elimination and exacerbates the accumulation of ROS. Studies have been conducted on PON 1 activity and revealed a reduction thereof in patients suffering from lung, breast, colorectal, oesophageal, and bladder cancer. Decreased concentrations of paraoxonase 1 have also been reported in EC, which confirms that the impairment of antioxidant mechanisms plays a role in the aetiopathogenesis of this cancer [79].

Neoplastic cells can increase the production of ROS to trigger the overactivity of the signalling pathways necessary for cellular transformation and neoplasia, whereas, in order to prevent cell death and maintain the oxidoreductive homeostasis, cancer cells can increase their own antioxidative capacity [51]. Due to the variable redox status of tumour cells, they become vulnerable to therapies aimed at modifying the ROS content. A survey study was conducted in the USA to analyse the frequency with which 410 EC patients and 395 healthy women consumed particular food products [80]. The cited authors concluded that the total consumption of phenols might decrease the risk of EC but found no evidence to support a connection between the consumption of any specific antioxidant and the incidence of EC. These results might suggest that the most important element of EC prophylaxis is bolstering the organism’s overall antioxidative defences. This protective system operates on three levels: (1) blocking the formation of ROS thanks to exogenous antioxidative enzymes, (2) interrupting free radical chain reactions, and (3) eliminating the results of ROS reactions and rebuilding the structure of damaged cells [81].

## 5. Diet-Dependent EC Comorbidities

Oestrogens play a key role in the development of type 1 EC. At the same time, such a dependence is not observed in type 2 EC cases [6]. In a study conducted on hamsters, it was demonstrated that simultaneous exposure to oestrogens and oxidative stress triggered the emergence of neoplasia [82]. The risk of type 1 EC increases if the woman already suffers from obesity, type 2 diabetes and/or insulin resistance, or metabolic syndrome [7]. The physiological markers of all those diseases include functional disorders of the antioxidative system and progressing inflammation [10,11,12].

### 5.1. Obesity

**Figure 3:**
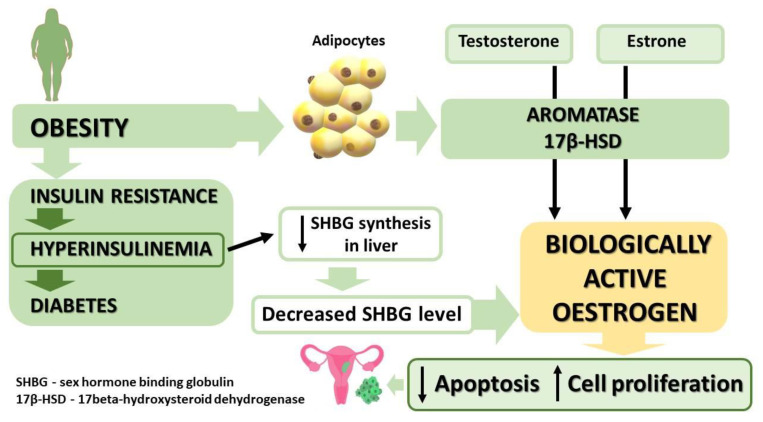
Mechanism of oncogenesis as mediated by active estrogen in hyperinsulinemia. ↑ increased, ↓ decreased.

Of all cancers, EC shows the strongest positive correlation with obesity, as evidenced in a study on endometrial samples collected during hysterectomy from women with correct body weight (BMI 20–25) and overweight or obese patients [83]. Every five-point increase in BMI corresponded to a 1.6-fold increase in the risk of contracting EC, which means that the risk is 10-times higher for patients with a BMI of 42 as compared to women with normal body weight. This is related to the increased accumulation of lipids in the adipose tissue, which in turn elevates the capacity of such tissue (particularly white fat) to synthesise oestrogens, thus increasing the levels of circulating oestrogens that may stimulate the development of cancer cells in the uterus [7] (Figure 3). In postmenopausal women, oestrogens are no longer produced by the ovaries but by the adipose tissue through the conversion of androstenedione to oestradiol by aromatase, with the participation of the cytochrome p450 enzyme complex [84]. The concentration of oestrogens is higher in the adipose tissue than in blood serum; hence, the tissue can also be a site of EC metastases [85]. Consequently, the elevated concentration of oestrogens increases the risk of EC in obese women. It is noteworthy that the adipose tissue of obese individuals is very active metabolically and endocranially as it produces numerous cytokines, such as the tumour necrosis factor-alpha (TNF-a), leptin, perilipin, and proinflammatory interleukins. Compared to women with normal body weight, obese women show increased proliferation of endometrial cells, as evidenced by the elevated expression of the Ki-67 marker and the increased phosphorylation of proliferative Akt signal proteins [83]. In a study conducted in a group of 193 asymptomatic obese women, the incidence of latent endometrial hyperplasia and EC was, respectively, 12% and 3% in postmenopausal women and 6% and 1% in premenopausal women [86]. It was demonstrated that the expression of the insulin-like growth factor-1 (IGF-1) in EC increases with the body mass index and affects the extent of pathological lesions [87]. Unlike other hormones secreted by adipocytes, the concentration of adiponectin is lower in obese patients. Moreover, elevated levels of leptin and low levels of adiponectin, which may serve as markers of insulin resistance, have also been associated with the increased risk of EC [88,89]. Adiponectin, produced by the adipose tissue, inhibits the synthesis and secretion of proinflammatory cytokines: tumour necrosis factor-alpha (TNF-α) and interleukins IL-1 and IL-6 in macrophages, inhibits the differentiation of monocyte precursors and stimulates the synthesis of proinflammatory cytokines in monocytes, macrophages, and dendric cells [90]. Moreover, it has been demonstrated that the concentration of adiponectin is more strongly correlated with insulin sensitivity and insulinemia than the content of white adipose tissue in the organism, which suggests that hypoadiponectinemia in obese patients with type 2 diabetes results from primary insulin resistance and/or hyperinsulinemia [91]. Adiponectin increases insulin sensitivity in the muscles and liver with the participation of the 5′adenosine monophosphate-activated protein kinase (AMPK) pathway, reducing the concentration of free fatty acids in the plasma and demonstrating anti-atheromatous properties [92].

It is believed that physical activity can reduce the risk of EC as, apart from the obvious benefit of reducing body mass, it lowers the concentration of oestradiol in the blood serum by increasing that of sex-hormone-binding globulin (SHBG)—a protein capable of binding oestradiol synthesised in the liver [93]. This impact on oestrogen metabolism may, at least to some extent, have beneficial effects, either directly or by reducing the organism’s fat deposits [7]. It is further believed that a sedentary lifestyle leading to obesity significantly increases the risk of insulin resistance, which in turn leads to the development of type 2 diabetes and related metabolic syndrome [7,94]. Regular, moderate physical activity improves the basic metabolism and maximum oxygen intake, which improves long-term metabolic efficiency and capacity, consequently reducing the blood serum concentration of insulin and insulin resistance and stimulating the synthesis of adiponectin [92,93]. Prospective studies conducted in several countries among morbidly obese patients undergoing bariatric surgery revealed that permanent weight loss reduced the incidence of cancer in women by 38% [89,95,96].

Systemic oxidative stress, caused by the impairment of the antioxidative defence system responsible for neutralising ROS, and chronic inflammation are among the primary markers of obesity, as shown in numerous human and animal studies [97,98]. Oxygenation and an incorrect protein structure lead to dysfunctions of adipocyte proteasomes, which in turn triggers the hyperactivation of protein kinase and insulin resistance mediated by oxidative stress affecting the endoplasmic reticulum in the liver, which is directly related to inflammation [99]. Oxidative stress is intensified with increasing BMI as a consequence of the antioxidative status impairment, shortage of autoantibodies against oxygenated LDL, increased concentrations of peroxides and uric acid, and an unfavourable lipid profile [100]. ROS production was observed to increase selectively in the adipose tissue of obese mice, which was accompanied by the elevated expression of NADPH oxidase and the reduced expression of antioxidative enzymes [101,102]. The exacerbation of oxidative stress in the accumulated fatty tissue is an early initiator of metabolic syndrome [101].

### 5.2. Type 2 Diabetes and Insulin Resistance

Chronic hyperglycaemia observed in uncontrolled diabetes intensifies the autooxidation of glucose and the formation of ROS, which in turn leads to micro- and macro-vascular dysfunctions and polyneuropathies caused by the organism’s endogenic antioxidative defences [103]. The oxidative stress that emerges under those circumstances triggers a range of cellular problems, e.g., structural deformation of lipids, denaturation of proteins, and the disruption of DNA replication mechanisms [12]. Indeed, the deformation of cellular organelles or even entire cells can take place [52].

The relationship between the incidence of EC and type 2 diabetes has been evidenced in numerous studies. Postmenopausal women diagnosed with diabetes are twice as likely to contract EC as non-diabetic women of the same age [104]. In patients suffering from type 2 diabetes, insulin resistance is often observed, which leads to hyperinsulinemia, a metabolic condition that increases the risk of EC (Figure 3) also in premenopausal women [105]. Similarly to healthy cells in the course of diabetes and insulin resistance, EC cells are affected by disruptions in the insulin and IGF-1 receptor pathways involved in the growth of tumour cells [106]. A higher expression of the IGF-1 receptor has been correlated with a lower pathological range of the disease and a higher rate of survival [87]. It is, therefore, hardly surprising that metformin—a traditional drug that increases susceptibility to insulin—has been in the sights of numerous researchers investigating new potential methods of treating EC. Metformin lowers blood levels of glucose by limiting the liver’s capacity to produce new glucose from glycogen and increases the uptake of blood glucose by muscle cells [107]. It has been demonstrated in numerous epidemiological studies that diabetic patients receiving metformin become significantly less likely to contract a range of carcinomas, including pancreatic, hepatic, colorectal, and breast cancer [108,109,110]. In the context of EC, metformin, directly and indirectly, inhibits the growth of tumour cells [111,112]. The direct mechanisms of metformin activity include inhibiting the signal pathways of LKB1-AMP-activated protein kinase-mTOR, PI3K-Akt, and the pathway related to IGF-1, which limits the proliferation and promotes the apoptosis of EC cells. As for the indirect mechanisms, metformin increases tissue sensitivity to insulin and reduces the concentration of insulin in circulation, which facilitates an increase in the concentration of circulating globulin capable of binding bloodstream oestrogens, whose elevated levels have been identified as an EC risk factor. Moreover, it has been demonstrated that hyperinsulinemia is positively correlated with metastasis to lymph nodes and a negative prognosis in EC patients [113].

Oxidative stress plays a significant part in the pathogenesis of diabetes while also exacerbating damage to the heart, liver, and other organs [114,115]. A study conducted on Sprague-Dawley rats revealed that oxidative stress was already present in renal glomeruli at an early stage of diabetes, where it induced an increase in haem oxygenase activity [116]. At the same time, the use of various exogenic antioxidants can effectively limit the symptoms and consequences of oxidative stress [116,117].

### 5.3. Metabolic Syndrome

Metabolic syndrome (a combination of obesity, hyperglycaemia, arterial hypertension, and hyperlipidaemia) has been identified as a major risk factor in a number of different cancers, including EC [118,119]. Although it is well established that all the constituent elements of metabolic syndrome are related to the development of EC, it remains unclear as to whether their effects are additive or synergistic. A cohort study conducted in Canada revealed that women diagnosed with both metabolic syndrome and EC had worse prognoses compared to patients without metabolic syndrome and were more likely to suffer a relapse [120]. In the cited study, of the 540 women who survived EC, 325 suffered from metabolic syndrome at the time of the diagnoses, and in 132, the disease relapsed after a time. Similarly, in a study by Rosato et al. [121], a direct relationship was reported between the respective elements of metabolic syndrome, except overweight, and the risk of endometrial cancer; the study was conducted in a group of 454 women with a history of EC. In the US, in women aged 65 or older, metabolic syndrome and its respective elements were found to increase the risk of EC [122]. Although there are no definitive data evidencing a relationship between arterial hypertension (an element of metabolic syndrome) and the development of tumours, a connection has been suggested between hypertension and increased cancer-related mortality, with a possible contribution of apoptosis inhibition [92]. The measurement of calcium levels in the blood serum has been recently suggested as a possible parameter when evaluating metabolic syndrome in EC [123]. Based on results obtained in a group of 200 patients, the cited authors concluded that the level of total calcium might provide a metabolic syndrome parameter more sensitive than lipids in cases of endometrioid EC.

A growing body of reports confirms that oxidative stress, chronic inflammation, and angiogenesis all play significant roles in the pathogenesis of metabolic syndrome. The increase in oxidative stress parameters observed in accumulated adipose tissue is an important pathogenetic mechanism of metabolic syndrome, as demonstrated in several human studies [124,125]. Moreover, oxidative stress is not only a consequence but also a cause and primary link in the pathogenetic chain of metabolic syndrome [126]. The mechanisms underlying the development of atherosclerosis, one of the symptoms of metabolic syndrome, also include oxidative stress and inflammation [127,128]. A study conducted in a group of 2049 individuals revealed that lower levels of carotenoids and vitamin C, coupled with elevated levels of vitamins A and E, uric acid, and oxidative stress parameters in the blood serum, were related to an increased likelihood of metabolic syndrome [129]. Strategies capable of limiting factors responsible for exacerbating the incidence of metabolic syndrome may also prove beneficial in terms of EC prevention.

## 6. Anti-Obesogenic, Antidiabetic, and Antioxidative Properties of Tea

**Figure 4:**
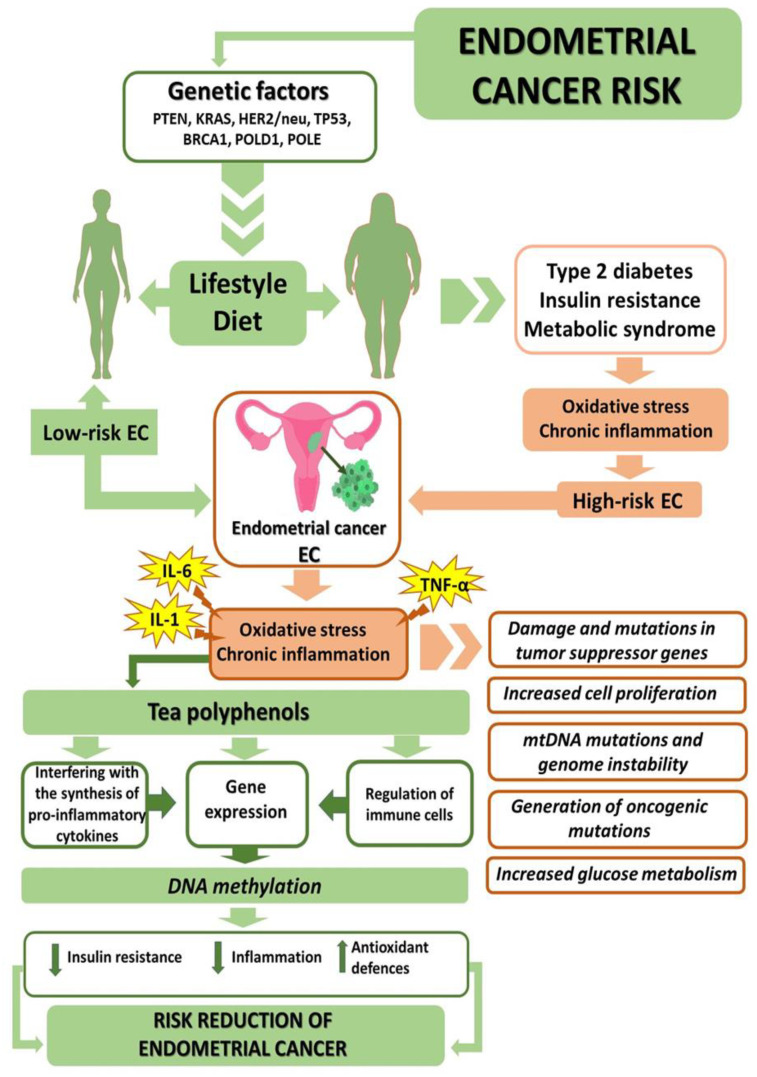
Beneficial effects of tea consumption on risk reduction in endometrial cancer. ↑ increased, ↓ decreased.

Dietary patterns and lifestyle habits are the main modifiable factors affecting the risk of cancer [7]. Maintaining the correct body weight, consuming healthy food, and engaging in regular physical exercise are all considered important aspects of cancer prevention. Morbidly obese women with EC are considerably more likely to die due to coexisting diseases such as cancer than their slimmer counterparts [130]. Obesity is a confirmed risk factor relevant to EC but also to the systemic oxidative stress caused by inflammation (Figure 4). It has been suggested that reactive oxygen species contribute to the carcinogenic process, including the stages of initiation, promotion, and progression. A study conducted on a group of 199 women revealed that the markers of oxidative stress were significantly correlated with EC as compared to patients not suffering from cancer [57]. Studies show that there is a significant correlation between elevated BMI and oxidative stress markers [100]. Hence, the biomarkers may be further investigated as potentially viable screening tools usable in patients at increased risk of contracting EC [89].

Metabolic diseases: obesity, type 2 diabetes, insulin resistance, and metabolic syndrome are diet-related factors that increase the risk of EC. Tea may help in EC indirectly by reducing the risk of these diseases or alleviating their symptoms.

### 6.1. Anti-Obesogenic, Antidiabetic, and Antioxidative Substances Present in Tea

Tea contains a variety of bioactive substances that can potentially facilitate weight loss: (1) by reducing appetite through the stimulation of noradrenaline synthesis and activation of the sympathetic nervous system, which creates the sensation of satiety; (2) increasing energy expenditure by lowering the metabolic rate; (3) stimulating lipid metabolism by activating the adrenergic receptor; (4) inhibiting the production of pancreatic lipase through covalent bonding with serine in the active section of the enzyme [131,132,133]. Active substances present in tea and showing properties that may facilitate weight loss include polyphenols, flavonoids, caffeine, caffeic acid, and chlorogenic acid [134].

Antidiabetic properties are associated primarily with catechins (mainly EGCG), gallic acid, caffeine, theaflavin, and polysaccharides. The compounds regulate the blood levels of glucose: (1) by lowering ROS concentrations; (2) inhibiting the activity of α-amylase and α-glucosidase, whereby the inhibitive activity depends on the number of hydroxy groups present in the compound; and (3) modulating the expression of proinflammatory cytokines that may reduce the glucose-induced secretion of insulin [135,136,137].

The phenolic compounds present in tea (primarily EGCG, quercetin, theaflavin, thearubigin, and tannic acid) have antioxidant properties thanks to their ability to: (1) limit the production of ROS by inhibiting the activity of oxidative enzymes and chelating trace elements; (2) sweep ROS; (3) increase the activity of endogenic antioxidants; and (4) donate an electron or hydrogen atom, which facilitates the neutralisation of singlet oxygen [45,52,138,139]. The highest antioxidative activity, resulting directly from the highest relative content of total polyphenols, has been observed for green and white tea [53,140]. Published research reveals a positive impact of consuming antioxidants in terms of improving the organism’s overall antioxidative status and lowering the risk of cancers, including EC.

### 6.2. Impact of Tea on Body Weight—Research Overview

The decrease in AMPK activity observed in the context of obesity may cause inflammation, while chronic inflammation, coupled with lowered antioxidative status, may, in turn, lead to diabetes and insulin resistance in obese patients [141]. Green tea supplementation improves the biomarkers of oxidative stress and modulates the levels of proinflammatory IL-6 in obese women [142]. The elevated levels of TNF-α emerging due to inflammation may induce insulin resistance. TNF-α is overexpressed in the adipose tissues of obese humans and animals alike, whereas obese mice without TNF-α or its receptor are reportedly immune to insulin resistance [143]. In a study on human adipocytes, it was observed that the caffeic and chlorogenic acid present in tea could induce lipolysis and stimulate the expression of AMPK [134]. Caffeine, naturally present in green tea, also impacts the activity of the sympathetic nervous system and can synergise with catechins, further increasing the energy expenditure and oxidation of fat [144,145]. The significance of caffeine was demonstrated in a study by Hsu et al. [146], where obese patients of both sexes suffering from type 2 diabetes received a caffeine-free green tea extract in an amount providing a daily dose of 856 mg of epigallocatechin gallate (EGCG). In the cited study, no significant impact of the caffeine-free extract on the subjects’ BMI was observed. Published results also evidence the anti-obesogenic activity of EGCG, most likely due to increased cellular production of ROS mediated by EGCG peroxidation, which leads to the activation of adenosine monophosphate-activated protein kinase responsible for inhibiting the expression of genes, enzyme proteins, and transcription factors involved in adipogenesis and lipogenesis [147]. Catechins inhibit the secretion of pancreatic and gastric lipases, thus limiting the emulsification and digestion of fats, as evidenced in a study by Hsu et al. [148] conducted on a group of 12 subjects consuming high-fat diets. It was demonstrated that green tea extract could effectively increase the levels of leptin and adiponectin, as well as reduce the level of ghrelin in obese women, lowering appetite and regulating lipid metabolism [149,150,151]. Green tea lowers the rate of intestinal absorption of lipids and proteins and activates a pathway reducing gluconeogenesis and fatty acid synthesis [152]. Another study conducted on a group of obese women revealed that green tea extract facilitated a significant loss of body weight and reduced visceral fat and waist size with no apparent side effects or adverse reactions [147,150,153,154]. Polyphenols isolated from green tea leaves, including L-epicatechin, epicatechin gallate (ECG), epigallocatechin (EGC) and EGCG, showed a strong capacity to inhibit pancreatic lipase [155]. It is noteworthy, however, that another study conducted by Hsu et al. [144] revealed no significant impact of 12-week green tea extract consumption on weight, adipose tissue mass, or the waist size of obese women, although a certain improvement in atherogenic parameters was reported as well as an increase in adiponectin levels and a decrease in ghrelin levels.

Tea has anti-obesogenic, antidiabetic, and antihyperlipidemic properties. Due to its easy and widespread availability, it may viably support obese patients in dealing with excessive weight and facilitate maintaining normal body mass by ones who have already succeeded in this battle. Naturally, successful weight loss remains dependent on the ability to maintain an overall healthy lifestyle, which poses a considerable challenge [131].

### 6.3. Antidiabetic Properties of Tea—Research Overview

Even though genetic predispositions are a major factor affecting the incidence of the disease, it is believed that environmental factors, including diet, can stimulate the initiation and progression of diabetes [12]. Based on preliminary research results, it can be concluded that by introducing tea into the diet, it is possible to alleviate some effects of oxidative stress and inflammation, which helps to limit their destructive impact on the organism of a diabetic patient, thus improving their quality of life, regardless of the particular type of diabetes. Furthermore, the elimination of inflammation can considerably weaken the immune response. It was demonstrated that green tea or green tea extract could reduce insulin resistance and improve glycaemic control [156]. In a European cohort study, it was observed that individuals drinking at least four cups of tea daily were 16% less likely to contract type 2 diabetes as compared to people not drinking tea [157]. Similar results were reported in cohort studies conducted in other countries [158,159,160]. A study conducted in China revealed that daily consumption of green tea led to a lower risk of contracting type 2 diabetes and lower mortality rates among diabetic patients [161]. Moreover, the daily consumption of tea also lowered the risk of microvascular complications in the course of diabetes. Tea contains polyphenols and caffeine, both of which show antidiabetic activity thanks to their antioxidative and anti-inflammatory properties [145]. Polyphenols also lower the blood concentration of glucose by inhibiting sugar transporters in the small intestine and increasing insulin susceptibility [162]. Catechins may be effective in controlling hyperglycaemia and preventing complications in diabetes by improving insulin susceptibility and reducing certain type 2 diabetes risk factors such as oxidative stress, dyslipidaemia, and obesity—as evidenced in both human and animal studies [163,164,165]. An in vitro experiment conducted on Caco-2 cells revealed that catechins are the dominant inhibitor of glucose uptake; moreover, particular tea catechins lowered the expression of the SGLT1 gene (sodium/glucose co-transporter-1) responsible for glucose absorption [162]. EGCG supplementation can be beneficial in terms of blood pressure, lipid profile, atherogenic factors, and oxidative stress, as demonstrated in a study involving type 2 diabetes patients [166]. EGCG effectively facilitated better glycaemic control and insulin susceptibility while at the same time improving the lipid profile and oxidative stress parameters in a rat model of type 2 diabetes [167]. Water extracts from various types of tea showed the capacity to inhibit the activity of α-glucosidase, aldose reductase, and advanced glycation end products thanks to the presence of polyphenols and catechins and the ability to promote glucose uptake [168]. The cited authors suggested that the regular consumption of tea, particularly of green and black varieties, may be helpful in preventing the progression of hyperglycaemia and vascular complications in diabetes. In turn, Han et al. [169] demonstrated that yellow tea, the least popular variety worldwide, has the strongest anti-hyperglycemic effects. Diabetic mice receiving a functional diet composed of corn-starch supplemented with tea polyphenols and EGCG showed a decreased fasting level of blood glucose as compared to mice not receiving polyphenols or EGCG; they also showed an improvement in terms of the intestinal microbiota profile [170]. Similar results were observed in diabetic rats receiving green tea enriched with catechin and inulin [171]. The administration of green tea to 12-week-old diabetic rats prevented the accumulation of glycogen in renal tubules by lowering the blood concentration of glucose, which prevented diabetic nephropathy [172].

Genetic predispositions may condition the organism’s response to the bioactive substances present in tea. For instance, it was demonstrated that carriers of the type 2 diabetes and obesity risk allele FTO-rs9939609 suffering from type 2 diabetes showed a better response to EGCG in terms of BMI improvement and diastolic blood pressure [173].

### 6.4. Impact of Tea on the Organism’s Antioxidative Parameters—Research Overview

The antioxidative properties of tea result from its high content of polyphenols such as catechins (including EGCG in green tea, quercetin, theaflavin, and thearubigin (in black tea), as well as tannic acid [45]. The total polyphenols correspond to between 25 and 35% of the dry solids present in tea leaves. Tea bolsters the organism’s antioxidative capacity—it enhances the activity of primary antioxidative enzymes such as GR, GPX, CAT, and glutathione transferase (GST), as evidenced in studies conducted on animals and with the participation of human subjects (Table 2 and Table 3).

It was demonstrated that the antioxidative potential in blood plasma increased by 34% after drinking green tea and by 29% after drinking black tea [216]. Wistar rats with streptozotocin-induced diabetes receiving a green tea extract showed an improvement in terms of antioxidant parameters as compared to the control [178]. The regular consumption of white tea by Wistar rats with the prediabetic condition improved the activity of antioxidative enzymes and total antioxidative capacity in the animas’ lungs and restored the values of protein nitration and lipid peroxidation [190]. A study on the antioxidant profile of rats exposed to a range of xenobiotics revealed a protective capacity of green, black, white, and red tea, as well as improvement in terms of the serum parameters of oxidative stress: SOD, CAT, GSH, GPX, and markers of lipid peroxidation (TBARS—thiobarbituric acid reactive substance) [53,177,179,180,181,197]. In Wistar rats receiving cardiotoxic doxorubicin, the use of a green tea extract triggered an increase in the activity of GPX, GR, GST, SOD, and CAT in the heart [182]. The consumption of green tea also improved antioxidative activity in human blood serum, and the effects were dose-dependent [217]. The general antioxidative capacity after consuming two cups of tea (one cup = 2.5 g of leaves/150 mL of water), as compared to drinking water, was 7% higher after 60 min and 6.2% higher after 120 min. Increasing the dosage to three cups of tea improved the antioxidative capacity of the blood serum by 12% after 60 min, and the effect persisted for at least 120 min after consuming the tea. Studies revealed that after the consumption of green tea, human blood plasma contained three times the amount of catechins when compared to drinking black tea [218]. An in vitro study with the use of human erythrocyte preparations revealed that green tea is characterised by higher antioxidative activity than white or black tea, as determined relative to the α- and γ-tocopherol levels in the analysed cells [219].

## 7. Tea as an Element of Prophylaxis and Pharmacotherapy Supplementation in Endometrial Cancer

Since disorders of the antioxidative system and progressing inflammation are physiological markers of EC, the primary form of supplementing pharmacological treatment should focus on providing a diet rich in substances facilitating the organism’s response to such conditions, particularly ones suitable for lifelong consumption. Tea is a beverage second only to water when it comes to worldwide popularity. It can be consumed without limitations, although it should be noted that some authors suggest that green tea can cause hepatitis or even acute hepatic failure. However, such cases are most likely due to individual oversensitivity [220]. Hepatotoxicity is probably caused by an enzymatic reaction (alcohol dehydrogenase, P450 cytochrome, mitochondrial enzyme), leading to cell damage and interference with biological response systems and metabolic reactions [221]. Contrary results were reported in rat studies where a green tea extract was shown to have hepatoprotective properties [53,222,223]. The antioxidative properties of tea are due primarily to its content of polyphenols, the highest, over 2000 mg in 1000 mL, in white and green tea, and approximately half that in black and red varieties [53]. Some authors suggest that polyphenols may be stronger antioxidants than, e.g., antioxidative vitamins [80]. Many mechanisms have been proposed to explain the anticancer properties of phenolic compounds, including in terms of their antioxidative and prooxidative activity as well as interference with cellular functions [47]. Phenols inhibit phase I enzymes, induce phase II enzymes, stimulate DNA repair, promote anti-inflammatory effects, induce the arrest of the cell cycle and apoptosis, and inhibit cell proliferation [47]. The antioxidative properties of phenolic compounds may stem from the following mechanisms: (1) neutralisation of free radicals; (2) reduction in ROS production by inhibiting certain enzymes or chelating trace metals involved in the formation of free radicals; and (3) enhancement of antioxidative defences [45,47]. The chemical structure of phenolic compounds is ideally suited to effective neutralisation of ROS due to the presence of hydroxy phenolic groups susceptible to hydrogen atom or electron donation, as well as an extensive coupled aromatic system facilitating the delocalisation of the unpaired electron 18 [45]. Polyphenols activate intracellular pathways, e.g., the arachnid acid-dependent pathway, nuclear transcription factor (NF-κB), mitogen-activated protein kinases (MAPK), or 3-kinase phosphatidylinositol/protein kinase B signal pathway (PI3K/Akt); they also stimulate epigenetic modulations regulating the organism’s immunological response [199].

Even though phenolic compounds are commonly consumed with food, it is only recently that their health benefits have drawn considerable attention, particularly due to their strong antioxidative potential and clear benefits in preventing the development of diseases associated with oxidative stress, such as cancer. The main polyphenol present in green tea, epigallocatechin-3 gallate (EGCG), has been identified as an effective agent in lowering the risk of EC, with the effect observed independently of other EC risk factors such as obesity or menopause [224]. In a preclinical study, EGCG inhibited the proliferation of EC cells and induced their death [225]. A study conducted on a human endometrial adenocarcinoma cell line (Ishikawa cells) revealed that EGCG inhibited the proliferation of cells and induced apoptosis via Akt and MAPK signals [226]. In the cited study, reduced expression of oestrogen and progesterone receptors, as well as the arrest of cells in the G0/G1 phase of the cell cycle, was also observed. In an animal study, EGCG inhibited angiogenesis [227,228], preventing the formation of new blood vessels needed to support large tumours. A study utilising a xenograft model revealed that the administration of a prodrug form of EGCG inhibited the proliferation of tumour cells and enhanced apoptosis in a time and dose-dependent manner, with the significant effect of reducing the formation of microvessels and therefore hindering blood flow to the xenografts [229]. Similar results were observed in a mouse model of ovary cancer [230], which suggests that such extracts may prove viable in the prevention of a variety of cancers. The protective potential of green tea exceeds that of black tea; it was suggested that drinking an additional two cups of green tea daily can reduce the risk of EC by 25% [231].

Tea does not cure but can support and strengthen the body. It has been proven to reduce the risk of diet-related factors that predispose to EC, such as obesity, diabetes, insulin resistance, and metabolic syndrome. However, the tea consumed as part of prevention must be used continuously and regularly. Only then is the likelihood of its effectiveness increased. Few studies are available linking tea consumption to the occurrence of EC. However, we believe that this issue should be looked into, given the proven health-promoting effects of tea.

## 8. Oxidative Stress and Epigenetic Modifications in Cancer Cells

Despite having an identical genome, human cells are exceedingly diverse when it comes to their forms and functions, which allows them to form phenotypically and functionally distinct tissues and organs. The specific profile of gene expression is the joint product of a number of epigenetic phenomena, such as DNA methylation, histone modification, and non-coding RNA that specifically regulates which gene is to be expressed at any given point [232]. DNA methylation can inhibit the expression of genes directly by preventing the attachment of transcription factors and RNA polymerase, as well as indirectly by recruiting MBD proteins (methyl-CpG-binding domain proteins) [233]. MBD proteins are the main candidates for recognising DNA methylation as they recruit remodelers of chromatin, histone deacetylase and methylase to methylated DNA associated with gene repression. Mutations within the MBD domains are observed in many diseases, including cancers, and lead to the loss of MBD binding specificity in the methylated locations and gene deregulation [234]. The mechanism through which DNA methylation is interpreted and influences genome regulation remains largely unknown.

Oxidative stress is a factor fuelling mutations and methylations, leading to changes at the m5C level (5-methylcytosine—the primary epigenetic DNA mark) and neoplastic evolution [235,236]. 8-oxo-deoxyguanosine (8-oxo-dG) is the specific marker of DNA damage induced by ROS, and its accumulation causes m5C to become the focal point for mutation [237]. Excessive ROS content exacerbates genome instability, which induces oncogenic mutations, loss of suppressor gene functions, hypoxia adaptation, and increased glucose metabolism [238]. ROS excess in tumour cells is quenched by the increased availability of antioxidative enzymatic pathways and non-enzymatic pathways in the same cells [239]. In the case of EC, the oxidative stress on DNA can contribute to neoplasia, but its intensification is most likely independent of the tumour’s progression [240]. A study conducted among 37 women undergoing hysterectomy revealed hypomethylation of DNA and statistically significantly reduced activity of DNA methyltransferase (DNMT1) in the tissues of uterine mucinous adenocarcinoma as compared to healthy endometrial tissue [241].

EC is an oestrogen-dependent cancer [242]. Oestrogens increase ROS tolerance in cells by stimulating the production of ROS and accelerating their elimination, which leads to carcinogenesis and the promotion of malignant transformation [243]. By promoting mutations in mitochondrial DNA (mtDNA) and damage to mitochondrial proteins, oestrogenic mitochondrial ROS affect energy metabolism and modulate the redox-sensitive proteins responsible for cell proliferation and anti-apoptosis [244]. On the other hand, oestrogens have simultaneous beneficial antioxidative effects that protect tumour cells against the potentially toxic influence of ROS [243]. Elevated ROS levels can induce cell death, which is why neoplastic cells adapt to oxidative stress by regulating intracellular antioxidative proteins to maintain a level of ROS sufficient to facilitate pro-tumorigenic signalling without causing cell death [245]. Since ROS are capable of triggering programmed cell death, Perillo et al. [239] referred to ROS generated by various metabolic pathways as “Trojan horses” in the context of tumour cell elimination. Certain molecular mechanisms exist that may prove useful in therapeutic strategies based on modulating ROS levels as part of cancer treatment.

**Figure 5:**
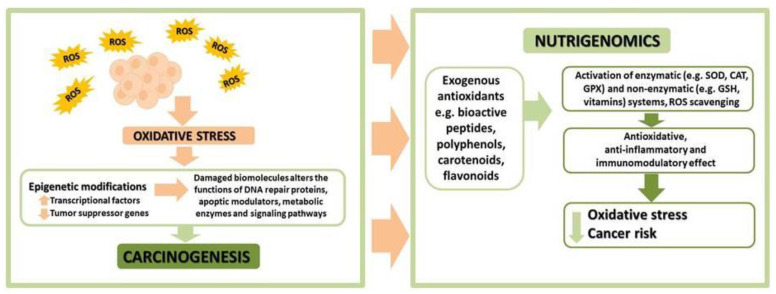
The role of oxidative stress in cancer and the influence of food components on the occurrence of oxidative stress. ROS—reactive oxygen species.

Epigenetic and genetic changes have long been considered two separate mechanisms involved in carcinogenesis [246]. It has been discovered, however, that there are many mutations affecting the genes that control the epigenome. Such mutations can potentially interfere with epigenetic processes, such as DNA methylation, histone modification, and nucleosome positioning, which disrupt the expression of genes and can be a factor contributing to tumour emergence. An impact of epigenesis on the induction of local mutations and exclusion of DNA repair functions has also been observed. Due to the mutual interactions between the genome and the epigenome, therapies entailing epigenetic regulation, for instance, nutrigenomics, can prove viable (Figure 5). Nutrigenomics is an area of research focusing on the impact of food ingredients on the expression of genes that may condition the incidence of various chronic diseases [247].

## 9. Perspectives—Tea as an Epigenetic Regulator in Endometrial Cancer Therapy

Oxidative stress promotes the mutation and methylation of DNA, which initiates carcinogenesis and promotes malignant transformation [235,236]. It has been well established that oxidative stress is a marker of EC; however, it remains to be determined whether increased oxidative stress is a cause or result of EC aetiopathogenesis. The phenomenon of oxidative stress in cancer cells may be explored in search of alternative strategies in cancer treatment that would entail supplying exogenic antioxidants to facilitate the endogenic cellular antioxidative defence systems. The reactivity of antioxidative agents towards particular oxidants can be very varied, which suggests the need to use a range of antioxidants simultaneously. Tea is a beverage rich in a variety of different antioxidants. For this reason, it is worth considering the promotion of tea consumption among individuals with genetic predispositions for EC, especially given its low cost, wide availability, generally beneficial effects on human health, and above all, its suitability for long-term use regardless of the patient’s age. It is difficult to conclusively determine which particular type of tea is the most beneficial in the context of carcinogenesis pharmacotherapy support, but the available literature suggests the highest activity of EGCG, a compound found in green tea. The particularly high antioxidative capacity of EGCG is due to its specific chemical structure containing as many as eight–OH groups [45]. Some authors mention problems related to the low bioavailability of EGCG, which is why research is currently conducted with a view to overcoming this barrier with the use of nanotechnologies involving encapsulation, liposomes, micelles, and nanoparticles [248]. Such methods may prove effective, although their applicability would likely be limited to individuals already suffering from EC as an element of diet therapy supplementing medical treatment. When it comes to prophylaxis in genetically predisposed patients, consuming EGCG in the form of tea seems to be a more viable and safer alternative.

In conclusion, tea does not cure but can support and strengthen the body. It has been proven to reduce the risk of diet-related factors that predispose to EC, such as obesity, diabetes, insulin resistance, and metabolic syndrome. However, the tea consumed as part of prevention must be used continuously and regularly.

## Figures and Tables

**Table 2:** Antioxidant effects of tea and tea active compounds—review of studies on laboratory animals.

Type/Form of Tea	Antioxidant Factor	Oxidative Stress Parameters	Target Sites	Animal Species	References
Green tea	Photoaged skin	↑ hydroxyproline; ↑ CAT; ↓ protein carbonyl	Serum, skin	Mice	[174]
Green tea	Lead acetate (0.4%) orally treated rats	↓ LPO; ↑ GSH-Px; ↑ SOD; ↑ CAT	Kidney	Sprague Dawley rats	[175]
Green tea	Lead acetate (0.4%) orally treated rats	↑ SOD; ↑ GST	Liver	Sprague Dawley rats	[176]
Green tea	High-sodium-diet (4 g NaCl/kg diet) induced hypertension	↑ TAC	Serum	Wistar rats	[177]
Green tea	Streptozotocin-induced diabetic rats	↓ MDA; ↑ GSH; ↑ SOD; ↑ CAT; ↑ GSH-Px	Liver, serum	Wistar rats	[178]
Green tea extract	Lead acetate (0.4%) orally treated rats	↓ LPO; ↑ GSH; ↑ SOD; ↑ GST	Serum	Sprague Dawley rats	[179]
Green tea extract	Atherogenic diet consisting of 33% sugar, 21% fat, and 3% cholesterol	↑ TAC; ↓ SOD; ↓ CAT; ↑ GSH; ↑ GSH-Px	Serum	Sprague Dawley rats	[180]
Green tea extract	Lead acetate (100 mg/kg b. w.) treated rats	↑ SOD; ↑ GSH-Px	Total brain	Albino rats	[181]
Green tea extract	Doxorubicin-induced cardiotoxicity	↑ GSH-Px; ↑ GR; ↑ GST; ↑ SOD; ↑ CAT	Heart	Wistar rats	[182]
Green tea extract	Chronic gasoline vapour inhalation	↑ CAT; ↑ SOD; ↑ GSH-Px; ↑ GST; ↑ total thiol; ↓ TBARS; ↓ AOPP	Brain	Male mice CD1 strain	[183]
Green tea extract	Nicotine—injected intraperitoneally (1 mg/kg body weight/day)	↑ SOD; ↑ CAT	Blood	Rats	[184]
Green tea infusion	0.4% lead acetate in distilled H_2_O orally	↓ LPO	Plasma, erythrocytes	Male rats	[184]
Green tea infusion	0.4% lead acetate in distilled H_2_O orally	↓ SOD; ↓ GST; ↓ GSH; ↓ NO; ↓ LPO	Liver, kidney, brain	Male rats	[185]
Green tea infusion	0.4% lead acetate orally for 6 weeks	↑ GST; ↑ GSH; ↑ SOD; ↑ TAC; ↓ LPO	Blood	Rats	[186]
Green tea infusion	0.4% lead acetate orally for 6 weeks	↑ GST; ↑ GSH; ↑ SOD; ↑ TAC; ↓ LPO	Brain	Rats	[186]
Aqueous extracts of rooibos tea	Chronic restraint or immobilization	↓ LPO; ↑ GSH ↑ GSH/GSSG	Blood	Rats	[187]
Aqueous extracts of rooibos tea	Chronic restraint or immobilization	↓ GR; ↑GSH-Px; ↓ SOD; ↓ CAT	Brain	Rats	[187]
Water extracts Pu-erh tea	High-fat diet	↓ MDA; ↓ NOS; ↑ SOD; ↑ CAT ↑ GSH-Px	Serum	Rats	[188]
White tea	Acute hypoxia and subsequent recovery juveniles	↓ SOD (especially Mn-SOD and CuZn-SOD isoforms); ↑ GSH-Px	Blood	*Sparus aurata*	[189]
White tea	Prediabetes rats	↑ SOD; ↑ CAT; ↑ GSH-Px	Lungs	Wistar rats	[190]
Green, black, red and white tea	Cadmium chloride (7 mg/kg feed) and lead acetate (50 mg/kg feed) orally	↑ SOD; ↑ CAT; ↑ GSH; ↑ GSH-Px	Lungs, brain, heart, liver, kidneys	Wistar rats	[53]
Catechin	Catechin—50 mg/kg or escitalopram—10 mg/kg orally	↑ CAT; ↑ SOD; ↓ GSH; ↓ MDA	Brain	Rats	[191]
EGCG	Fluoride 25 mg/kg/bw orally	↑ CAT; ↑ SOD; ↑ GSH-Px; ↑ GSH; ↑ GST; ↑ GR; ↓ ROS; ↓ TBARS; ↓ NO; ↑ Vit. C	Brain	Wistar rats	[192]
EGCG	Effect of electromagnetic radiation (frequency 900 MHz modulated at 217 Hz, power density 0.02 mW/cm^2^, SAR 1.245 W/kg)	↑GSH, ↑CAT, ↑GSH-Px; ↑ SOD	Brain	Rats	[193]
EGCG	Azoxymethane—induced colon carcinogenesis (5 mg/kg body weight)	↓ NOS 2, ↓ COX; ↓ NO	Colonic tissues	Mice	[194]
EGCG	7,12-dimethylbenz[a]-anthracene and 12-O-tetradecanoylphorbol-13-acetate-promoted skin tumorigenesis	↓ NOS; ↓ COX	Skin	Mice	[195]
Green tea tannins	Sodium arsenite (100 mg/kg/day) orally for 28 days	↑ GSH; ↑ GSH-Px; ↑ SOD; ↑ ALAD; ↑ NOx; ↓ TBARS	Liver, kidneys	Sprague Dawley rats	[196]
Tannic acid	Cadmium chloride (7 mg/kg feed) and lead acetate (50 mg/kg feed) orally	↑ SOD; ↑ CAT	Total brain	Wistar rats	[197]
Tannic acid	Lead acetate (50 mg/kg body weight) for three times a week for two weeks	↓ LPO; ↑ GSH; ↑ GST; ↑ GSH-Px; ↑ SOD; ↑ CAT	Brain	Wistar rats	[198]
Caffeine	Cadmium chloride (5 mg/kg)	↓ LPO; ↓ ROS, ↑ Nrf-2; ↑ HO-1	Brain	C57BL/6N mice	[199]
Green tea	Azathioprine-induced liver damage	↓ GSH; ↓ CAT; ↓ GSH-Px	Liver	Rats	[200]
Tea polyphenols	Gastric cancer	↑ GSH-Px; ↑ SOD; ↓ MDA	Thymus, spleen	Mice	[201]
Tea polyphenols	Diethylnitrosamine/phenobarbital-induced hepatocarcinogenesis	↑ TAC; ↑GSH-Px	Liver	Sprague Dawley rats	[202]
Tea polysaccharides	Gastric cancer	↓ MDA; ↑ SOD; ↑ CAT; ↑ GSH-Px	Tissue	Mice	[203]
Tea polysaccharides	Exhausting training	↑ SOD; ↑ CAT; ↑ GSH-Px; ↓ MDA	Serum, liver, kinder	Mice	[204]
Tea polysaccharides	Carbon tetrachloride-induced liver injury	↑ SOD; ↑ GSH-Px; ↓ MDA	Liver	Mice	[205]

↑—increased concentration or activity compared to treated group; ↓—decreased or inhibited concentration or activity compared to treated group; ALAD—delta-aminolevulinic acid dehydratase; AOPP—advanced oxidation protein products; CAT—catalase; COX—cyclooxygenase; CuZn-SOD—copper and zinc-containing superoxide dismutase; EGCG—epigallocatechin gallate; GSH—glutathione; GSH-Px—glutathione peroxidase; GR—glutathione reductase; GST—glutathione-S-transferase; GSSG—oxidized glutathione; HO-1—hemeoxygenase-1; LPO—lipid peroxidation; LOOH—lipid hydroperoxides; MDA—malondialdehyde; Mn-SOD—manganese-containing superoxide dismutase; MPO—myeloperoxidase; NO—nitric oxide; NOS—nitric oxide synthase; NOS 2—nitric oxide synthase 2; NOx—index of nitrite/nitrate; Nrf-2—nuclear factor-2 erythroid-2; ROS—reactive oxygen species; SOD—superoxide dismutase; TAC—total antioxidant capacity; TBARS—thiobarbituric acid reactive substances; Vit. C—vitamin C.

**Table 3:** The effect of tea and tea active compounds on oxidative stress-related alterations in human.

Type/Form of Tea	Antioxidant Factor	Oxidative Stress Parameters	Place of Collection	References
Rooibos	Risk for cardiovascular disease	↓ CDs; ↓ TBARS, ↑ GSH, ↑ GSH/GSSG	Blood	[206]
Oolong tea	Hypercholesterolemia	↑ NEAC	Serum, urine	[207]
Green tea	Healthy subjects	↑ hOGG1; ↑ HMOX-1	Blood	[208]
Green tea	Oxidation-induced DNA damage and redox-sensitive cytoprotective factors in type 2 diabetes patients	↑ TAC; ↑ GSH	Plasma	[209]
Green tea	Metabolic syndrome	↑ GSH	Blood	[210]
Black tea theaflavins	Neuronal apoptosis	↑ SOD; ↑ CAT	PC12 cells	[211]
EGCG	Hydrogen peroxide-induced injury in human dermal fibroblasts	↑ GSH-Px; ↑ SOD; ↓ MDA	Skin	[212]
EGCG	Hepatic stellate cell	↑ GSH	Liver	[213]
EGCG	Induced cancer cell death	↓ Trx/TrxR	Liver	[214]
EGCG	Cancer biopsies and HeLa cell line with hydrogen peroxide	↓ GSH; ↓ SOD	Tissue	[215]

↑—increased concentration or activity compared to treated group; ↓—decreased or inhibited concentration or activity compared to treated group; CAT—catalase; CDs—conjugated dienes; EGCG—epigallocatechin gallate; GSH—glutathione; GSH-Px—glutathione peroxidase; GSSG—oxidized glutathione; HMOX-1—heme oxygenase; hOGG1—8-oxoguanine glycosylase; NEAC—non enzymatic antioxidant capacity; SOD—superoxide dismutase; TAC—total antioxidant capacity; TBARS—thiobarbituric acid reactive substances; Trx—thioredoxin; TrxR—thioredoxin reductase; MDA - malondialdehyde.

## Data Availability

Data sharing not applicable.
